# *Schizophyllum Commune* a Causative Agent of Fungal Sinusitis: A Case Report

**DOI:** 10.1155/2011/821259

**Published:** 2011-09-12

**Authors:** T. Premamalini, B. T. Ambujavalli, S. Anitha, L. Somu, Anupma J. Kindo

**Affiliations:** ^1^Department of Microbiology, Sri Ramachandra Medical College and Research Institute, Sri Ramachandra University, Porur Chennai 600116, India; ^2^Department of Otolaryngology, Sri Ramachandra Medical College and Research Institute, Sri Ramachandra University, Porur Chennai 600116, India

## Abstract

We present a case of maxillary sinusitis caused by *Schizophyllum commune*, in a 50-year-old female. The patient presented with nasal obstruction, purulent nasal discharge from right side of the nose, cough, headache, and sneezing. Computed tomography revealed extensive opacity of the right maxillary sinus as well as erosion of the nasal wall and maxillary bone. Functional endoscopic sinus surgery was done, and fungal debris present on right side of the maxillary sinus was removed and sent to laboratory. Potassium hydroxide (KOH) examination of the nasal discharge showed hyaline, septate hyphae. Primary isolation on Sabouraud's dextrose agar (SDA) yielded a white woolly mould. Banana peel culture after 8 weeks showed macroscopically visible fan-shaped fruiting bodies. Lactophenol cotton blue (LPCB) mount of the same revealed hyaline septate hyphae, often with clamp connections. Identification was confirmed by the presence of clamp connections formed on the hyphae and by vegetative compatibility with known isolates.

## 1. Introduction 

*Schizophyllum commune* is a mould of phylum basidiomycota, Schizophyllaceae family, with worldwide distribution that colonizes diverse trees and rotting wood [1]. Infections originating from this fungus are rare in humans. Diverse clinical cases include chronic or allergic sinusitis, pulmonary disease, ulcerative lesions of the palate, atypical meningitis, cerebral abscess, and possible onychomycosis, which can occur in immunocompetent and immunocompromised individuals [2, 3]. Infective propagules in this fungus are air transported, thus, most frequently compromising the paranasal sinuses [1]; hence, the most common affliction is sinusitis that presents three clinical manifestations: allergy, chronic, noninvasive, and invasive (acute or chronic) [4]. In the laboratory, *S. commune* grows with relative ease in culture media that do not contain cycloheximide; due to this characteristic, it may be suspected that it is an environmental contaminant. But upon positive direct examination and isolation of this fungus, it is necessary to carry out repeated cultures [5]. The low frequency of reports on pathologies caused by this fungus motivated the presentation of the current case.

## 2. Case Presentation

A 50-year-old female, with history of allergic rhinitis, presented with nasal obstruction, purulent nasal discharge from right side, cough, headache, and sneezing. She had completed a 10-day course of doxycycline with minimal symptomatic improvement. She was not a known case of diabetes, hypertension, bronchial asthma, or pulmonary tuberculosis. There was no history of previous surgery, facial trauma, distant travel, or drug abuse. On physical examination, a purulent discharge was noted in the right middle meatus. The left middle meatus was clear. The remaining examination of the head and neck revealed no significant findings. Her hemoglobin was 15.3 g/liter, her hematocrit was 0.45, and the leukocyte count was 7.4 × 10^9^/liter with a normal leukocyte differential. The chest X-ray was unremarkable. A bone density-computed tomography revealed extensive opacity of the right maxillary sinus as well as erosion of the nasal wall and maxillary bone. The patient underwent right-sided functional endoscopic sinus surgery with opening of the right maxillary sinus. Thick mucus was present in the right maxillary sinus. The material was removed and sent for culture and histological examination. No antifungal agents were prescribed at that point in time. After 5 days, the patient came with complaint of bleeding from the right nostril. Right nostril was packed using a roller gauze and left nostril with IVALON nasal pack. Clots were visualized in B/L maxillary antrum using 30-degree scope, which were suctioned out. Mucosa was found to be healthy. No active anterior or posterior nasal bleeding. Patient was stable and asymptomatic and discharged.

**Figure 1:**
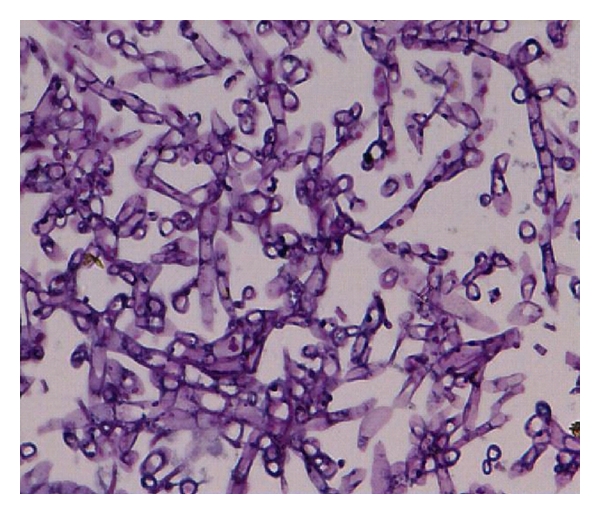
Periodic Acid Schiff (PAS) stain shows septate, fungal hyphae (Magnification 40x).

**Figure 2:**
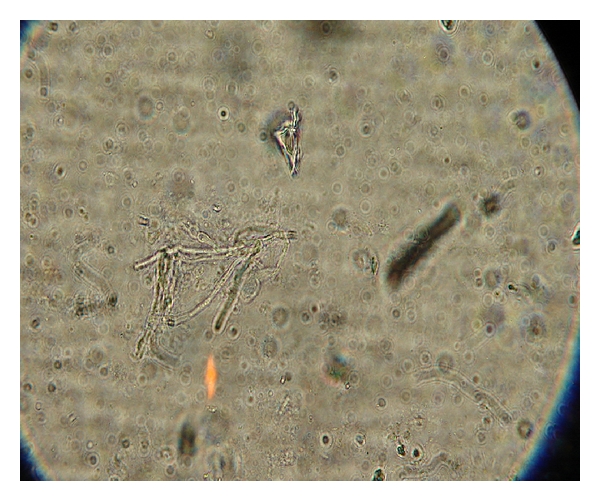
10% KOH mount shows hyaline septate hyphae. (Magnification 40x).

**Figure 3:**
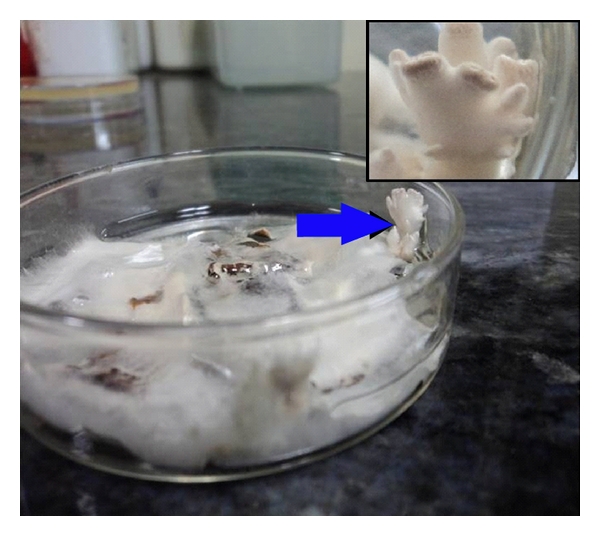
Fan-shaped fruiting bodies in banana peel culture (basidiocarps).

**Figure 4:**
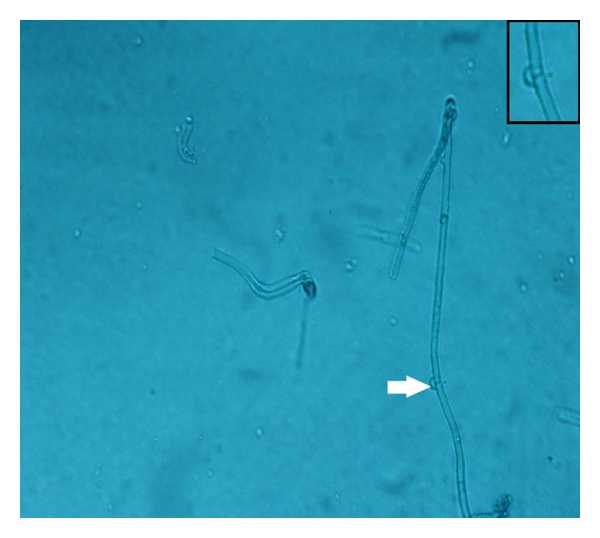
LPCB mount shows hyaline septate hyphae, with clamp connections, seen in the inset (Magnification 40x).

Specimens sent for histological studies showed extensive eosinophilic infiltration with Charcot-Leyden crystals. There was no tissue invasion. Examination after Periodic Acid Schiff (PAS) staining showed septate, nondichotomously branching fungal hyphae (Figure 1). Direct microscopy of a KOH preparation of the surgical specimen showed the presence of hyaline septate hyphae (Figure 2). Culture at 27°C and 37°C on Sabouraud's dextrose agar containing gentamicin yielded a rapidly growing, white to pale buff, densely woolly fungus with a pale-brown reverse. It produced a strong disagreeable odor. There was no growth on medium containing cycloheximide. No bacteria were isolated under aerobic or anaerobic conditions. Microscopic examination of the mold showed hyphae of various widths. The isolate was held for several months and subcultured on different media, but it failed to sporulate under any conditions. Banana peel culture after 8 weeks showed macroscopically visible fan-shaped fruiting bodies (Figure 3). LPCB mount of the same revealed hyaline septate hyphae, often with clamp connections or lateral pegs (Figure 4, inset). Identification was confirmed by the presence of clamp connections formed on the hyphae and by vegetative compatibility with known isolates. Further study showed the isolate to be tolerant of benomyl (2 *μ*g/mL), consistent with this basidiomycete [6].

## 3. Discussion

*S. commune* is a common invader of rotten wood. It occurs worldwide on a wide range of dead deciduous trees and uncommonly on vegetation such as hay, where its fan-shaped basidiocarps are easily identified [6, 7]. Although *S. commune* is ubiquitous in nature, there are only rare reports of its association with human infections [8]. Animal studies have shown that *S. commune* is a fungal agent of relatively low virulence causing a progressive low-grade infection, with death occurring in some very young animals, and in which the progress of infection is influenced by the age of the host, the size of the inoculum, and prior treatment with an immunosuppressive agent [8, 9]. Well-documented cases of *S. commune *infections include allergic bronchopulmonary disease, fungus ball in the lung, repeated isolation from the sputum of a patient with chronic lung disease, ulcerative lesions of the hard palate, and a nail infection. The isolation of *S*.* commune *from cerebrospinal fluid in a patient manifesting signs of atypical meningitis has been reported [10].

Fungal sinusitis is a rare entity that has increased in recent years in immunocompetent individuals. It reveals three clinical manifestations: allergic, chronic, noninvasive, and invasive. The allergic manifestation involves immunocompetent patients, and it is characterized by allergic mucin with eosinophils and Charcot-Leyden crystals, as well as increased serum IgE. The chronic noninvasive manifestation affects immunocompetent individuals and does not cause mucosal or blood vessel invasion. The invasive manifestation occurs in immunosuppressed individuals with tissue and vascular invasion [4].

The incidence of fungal sinusitis, particularly in immunocompetent patients, appears to be increasing [11, 12]. Although many cases of sinusitis have been attributed to the genus *Aspergillus*, several reports document the emerging role of *S. commune* in chronic or allergic sinusitis has been associated [4, 13–16].

*S. commune* is emerging as an important agent of sinusitis. Clinicians should include this fungus in the differential diagnosis of patients with chronic sinusitis refractory to standard therapy. It is likely that infections caused by *S. commune* are misdiagnosed or are not recognized because of clinicians' lack of familiarity with this fungus and the inability of many laboratories to identify this basidiomycete. Nevertheless, there are increasing numbers of reports of infections caused by *S. commune* in both immunocompetent and immunocompromised hosts. In immunocompromised patients, *S*.* commune* can even cause invasive infections like brain abscesses which can be fatal [10]. 

The optimal approach to the management of infections caused by *S. commune *is uncertain. The utility of the azoles in treating *S. commune *infections is not known. One patient with allergic bronchopulmonary infection was treated with itraconazole for 10 months without clinical or radiological improvement [17].

Amphotericin B is active in vitro against *S. commune*. A child with ulceration of the hard and soft palates was cured by amphotericin B therapy alone [18]. With sinusitis or invasive disease, surgical drainage or debridement and antifungal therapy would seem to be the approach of choice. The type, dose, and duration of antifungal therapy remain uncertain [10].

Clinical laboratories are familiar with the diagnostic features of the common fungal pathogens; however, the presence of clamp connections and hyphal tubercles may be overlooked, resulting in misidentification. Any white, nonsporulating mould that grows on routine mycologic media and that is inhibited by cycloheximide should be carefully studied as a possible *S. commune *isolate before being discarded as a sterile contaminant [2].

Thus, the isolation and accurate identification of the fungus are important in making the correct diagnosis of *S. commune* infection.

Dikaryotic isolates of *S. commune* are fairly readily recognized due to the presence of spicules and clamp connections on the hyphae and the development of fruiting bodies producing basidiospores. However, dikaryotic isolates may fail to fruit in the dark or on certain media or may lose their fruiting ability [6]. Clinical laboratories should not overlook basidiomycetes as potential opportunistic pathogens. Any white, rapidly growing, sterile isolate with septate hyaline hyphae should be suspected as *S. commune* if (i) it grows well at 37°C; (ii) it forms a dense, tough (i.e., difficult to cut), woolly colony; (iii) it is susceptible to cycloheximide (400 *μ*g/mL); (iv) it tolerates benomyl (2 *μ*g/mL) [6]; (v) it has a pronounced and disagreeable odor [2].

Therefore, a knowledge about *S. commune* will help the clinicians and microbiologists to correctly identify this fungus, so that a better understanding of its role in clinical disease will emerge. Correct diagnosis will also avoid nonspecific treatment, chronicity of illness, or complication to patients. 

The patient under discussion was treated with surgery alone as she was not willing for any antifungal therapy; she is on followup and is clinically asymptomatic at present.
